# Precision engineering of human cytomegalovirus without BAC constraints: a Sendai virus-delivered CRISPR/Cas9 approach

**DOI:** 10.1099/jgv.0.002126

**Published:** 2025-07-15

**Authors:** Jillian C. Carmichael, Christian S. Stevens, Kristina E. Atanasoff, Shreyas Kowdle, Rebecca A. Reis, Domenico Tortorella, Benhur Lee

**Affiliations:** 1 Department of Microbiology, Icahn School of Medicine at Mount Sinai, New York, NY 10029, USA

**Keywords:** bacterial artificial chromosome (BAC), CRISPR-Cas gene editing, human cytomegalovirus, virus entry

## Abstract

Human cytomegalovirus (HCMV) genetic manipulation traditionally relies on bacterial artificial chromosome (BAC) recombineering, necessitated by its large ~236 kb genome. This approach is limited by the scarcity of HCMV strains engineered into BACs and often requires the deletion of ‘non-essential’ genes to accommodate the BAC cassette. We developed a novel approach using temperature-sensitive Sendai virus (SeV) vectors to deliver CRISPR/Cas9 for targeted HCMV genome editing without these constraints. This system achieves high editing efficiency (80–90%) in fibroblasts, epithelial cells and endothelial cells without BAC intermediates. As proof of principle, we targeted the HCMV (TB40/E strain) pentamer complex (PC) genes UL128 and UL130, crucial for viral entry into non-fibroblast cells. Edited viruses showed significantly reduced infectivity in epithelial cells, confirming functional disruption of the PC. Plaque purification yielded isogenic clones with phenotypes comparable to AD169, a naturally PC-deficient strain. Furthermore, multiplexed editing created precise 663 bp deletions in over 60% of viral genomes. Importantly, this method enables HCMV editing in physiologically relevant cell types without fibroblast passaging, which typically introduces mutations. This SeV-Cas9 system represents a significant advancement for studying HCMV biology in diverse cell types.

## Data Availability

The sequence of the parental *ts* SeV-Cas9 vector has been deposited into GenBank (accession PV665661).

## Introduction

Human cytomegalovirus (HCMV) is a ubiquitous herpesvirus that infects an estimated 85% of adults worldwide [1]. While generally asymptomatic in healthy people, HCMV infection can cause severe disease in immunocompromised individuals, such as transplant recipients and people with human immunodeficiency virus/acquired immunodeficiency syndrome [2,3]. The virus can also be transmitted across the placenta during pregnancy, resulting in congenital HCMV, a leading cause of sensorineural hearing loss and developmental disability [4]. Upon infection, HCMV exhibits broad cell tropism in the host and establishes a lifelong latency, with CD14+ monocytes and CD34+ haematopoietic stem cells (HSCs) serving as reservoirs for the virus [5,6]. Currently, there is no FDA-approved vaccine or cure for HCMV [7]. Thus, the development of novel strategies to study HCMV biology and latency remains crucial for understanding the virus–host interactions and designing targeted therapeutic interventions.

Current genetic engineering techniques for herpesviruses like HCMV rely on bacterial artificial chromosome (BAC) recombineering, which allows for the manipulation of large viral genomes [8]. However, this method is constrained by the limited availability of isolated HCMV strains that contain the BAC cassette needed for recombineering [9]. Furthermore, many HCMV BAC constructs were constructed by inserting the BAC machinery in place of non-essential HCMV genes [10]. While these genes are not important for viral replication in fibroblasts, they can play essential roles for cell tropism, cellular metabolism, pathogenesis or latency, and removing them from the HCMV BACs prevents their study [11]. Finally, generating infectious virus from BACs requires the prolonged passaging of the virus in fibroblasts or epithelial cells, where it is well documented that HCMV can acquire mutations [12,13]. These restrictions have limited the study of HCMV latency, cell tropism and host pathogenesis, necessitating the development of alternative viral engineering approaches.

Sendai virus (SeV) is a murine paramyxovirus that has emerged as a unique viral vector for delivering gene editing technologies. Since paramyxoviruses contain a non-segmented negative-sense RNA genome and replicate entirely in the cytoplasm, the risk for unintentional viral integration into the host genome is minimal [14]. Additionally, SeV easily infects a majority of cell types including fibroblasts, epithelial cells, monocytes and CD34+ HSCs as it uses the ubiquitous sialic acid as its entry receptor [15]. Furthermore, the pleomorphic virion structure of SeV means the vector tolerates larger insertions into its genome than adeno-associated virus (AAV) vectors, allowing for the delivery of more varied transgenes [16,17]. An optimized reverse genetic system developed for paramyxoviruses has facilitated the cloning and rescuing of recombinant SeV vectors with various transgenes [18]. These features position SeV’s use as a flexible viral vector with broad applications.

Our lab previously developed a SeV vector containing Cas9 and a guide RNA (gRNA) cassette for highly efficient gene editing in primary human cells including monocytes and HSCs [19,20]. In this study, we engineered a temperature-sensitive (*ts*) SeV-based CRISPR/Cas9 delivery system to target HCMV viral DNA for editing in HCMV-infected cells. By leveraging the unique properties of SeV as a vector, this approach circumvents the limitations associated with BAC recombineering and enables targeted manipulation of the HCMV genome in a variety of cell types including fibroblasts, human umbilical vein endothelial cells (HUVECs) and epithelial cells (ARPE-19). As a proof of principle, we targeted the HCMV pentamer complex (PC) for editing with SeV-Cas9 in the aforementioned cell types. Consisting of five viral proteins (gH/gL/UL128/UL130/UL131A), the HCMV PC is crucial for viral entry into epithelial cells, endothelial cells and monocytes [21,24]. By disrupting the PC with our SeV-Cas9 vector, we successfully prevented viral entry into these non-fibroblast cell types and showed that we can edit HCMV genomes in fibroblasts and other cell types relevant to HCMV pathogenesis. This new methodology for creating recombinant HCMV will allow for more directed studies of HCMV latency, cell tropism and manipulation of cell processes and will aid in the development of innovative therapeutic strategies to combat HCMV infection.

## Methods

Key resources including (1) antibodies; (2) bacterial and virus strains; (3) chemicals, peptides and recombinant proteins; (4) chemicals, peptides and recombinant proteins; (5) critical commercial assays and kits; (6) cell lines; (7) recombinant DNA and primers; and (8) software are listed in Table 1.

**Table 1.** Key resources

Reagent or resource	Source	Identifier
**Antibodies**		
HCMV IE1/IE2 rabbit polyclonal	PMID: 35468974	n/a*
HCMV UL128 rabbit polyclonal antibody	This article	n/a
HCMV UL130 rabbit polyclonal antibody	This article	n/a
HCMV gH mAb 11D3	PMID: 35468974	n/a
HCMV gL rabbit polyclonal antibody	PMID: 27966523	n/a
Goat anti-Rabbit IgG (H + L ) Cross-Adsorbed Secondary Antibody, Alexa Fluor^tm^ 488	ThermoFisher	#A-11008
Goat anti-Rabbit IgG (H + L ) Highly Cross-Adsorbed Secondary Antibody, Alexa Fluor^tm^ Plus 647	ThermoFisher	#A32733
Donkey anti-rabbit HRP	Southern Biotech	#6441–05
**Bacterial and virus strains**		
HCMV TB40/E	PMID: 10580048	n/a
HCMV AD169	ATCC	VR-538
*ts* SeV-Cas9 gmCherry	PMID: 39494910	n/a
*ts* SeV-Cas9 UL128 g1	This article	n/a
*ts* SeV-Cas9 UL128 g2	This article	n/a
*ts* SeV-Cas9 UL130 g1	This article	n/a
*ts* SeV-Cas9 UL128 g2	This article	n/a
*ts* SeV-Cas9 US2	This article	n/a
Max Efficiency Stbl2 competent cells	ThermoFisher	10268019
Stellar competent cells (*Escherichia coli* HST08 strain)	Takara Bio	636766
**Chemicals, peptides and recombinant proteins**		
Dulbecco’s Modified Eagle Medium	ThermoFisher	11995-065
Eagle’s Minimum Essential Medium (EMEM)	ATCC	30-2003
DMEM/F-12 (Dulbecco's Modified Eagle Medium/Nutrient Mixture F-12) 1:1 medium	ThermoFisher	11320-033
EGM-2 (Endothelial Cell Growth Medium 2) Bullet Kit	Lonza	#CC-3162
Lebovitz’s L-15 media	ThermoFisher	11415-064
Opti-MEM reduced sera media	ThermoFisher	31985-070
Lipofectamine LTX and PLUS reagent	ThermoFisher	15338100
Penicillin-streptomycin	ThermoFisher	15140-122
0.25% trypsin-EDTA	ThermoFisher	2500-056
DPBS (Dulbecco's Phosphate Buffered Saline)	ThermoFisher	14190-144
UltraPure 0.5M EDTA	Invitrogen	15575-038
InFusion HD Cloning Kit	Takara Bio	639650
Cytogam	CSL Behring	#DC 49591-532-51
Ampure XP Beads	Beckman Coulter	A63881
16% paraformaldehyde solution	Electron Microscopy Sciences	15710-S
10% normal goat serum	Life Technologies	50062Z
Nonidet P40 Substitute	Sigma	74385
Triton™ X-100	Sigma	T8787
Protease Inhibitor Cocktail Tablets	Roche	04693132001
Pierce Protein A/G Beads	ThermoScientific	88803
Intercept Blocking Buffer	Li-Cor	927–70001
Mini-Protean TGX 4–15% precast gels	Bio-Rad	#456–1083
Protein assay dye reagent concentrate	Bio-Rad	#5000006
Immun-Blot LF PVDF membrane	Bio-Rad	#162–0260
10X Tris/glycine/SDS buffer	Bio-Rad	#1610772
Q5 high-fidelity DNA polymerase	NEB	M0491S
pGEM-T Easy Vector Systems	Promega	A1360
SeaPlaque™ GTG™ Agarose	Lonza	50115
**Critical commercial assays**		
MycoAlert Plus Mycoplasma Detection Kit	Lonza	#LT07-710
PureLink™ Viral RNA/DNA Mini Kit	ThermoFisher	12280050
PureLink™ HiPure Plasmid Filter Midiprep Kit	ThermoFisher	K210015
Gel and PCR clean-up	Macherey-Nagel	740609.250
SensiFast SYBR and Fluorescein Kit	Meridan Bioscience	BIO-96005
Ligation Sequencing Kit	Oxford Nanopore Technologies	SQK-LSK 109
Native Barcoding Expansion 1–12 (PCR-free)	Oxford Nanopore Technologies	EXP-NBD104
NEBNext Companion Module for Oxford Nanopore Technologies Ligation Sequencing	NEB	E7180S
Qubit™ dsDNA BR Assay Kit	ThermoFisher	Q32853
SpotON Flow Cell MK1 R9 Version	Oxford Nanopore Technologies	FLO-MIN106
**Experimental models: cell lines**		
MRC-5 cells	ATCC	CCL-171
ARPE-19 cells	ATCC	CRL-2302
Vero cells	ATCC	CCL-81
BSR-T7 cells	PMID: 9847328	n/a
HUVECs	PMID: 39494910	n/a
U373-MG gH/gL/UL128 cells	PMID: 27966523	n/a
**Oligonucleotides**		
*Primers used in this study listed in Table S1*		
**Recombinant DNA**		
Plasmid: T7 opt in pCAGGS	Addgene	Plasmid #65974
Plasmid: T7-SeV-N	PMID: 28405630	n/a
Plasmid: T7-SeV-P	PMID: 28405630	n/a
Plasmid: T7-SeV-L	PMID: 28405630	n/a
Plasmid: HCMV UL83 in pGEM-T	This article	n/a
*ts* SeV-Cas9 gmCherry	PMID: 39494910	n/a
*ts* SeV-Cas9 UL128 g1	This article	n/a
*ts* SeV-Cas9 UL128 g2	This article	n/a
*ts* SeV-Cas9 UL130 g1	This article	n/a
*ts* SeV-Cas9 UL128 g2	This article	n/a
*ts* SeV-Cas9 US2	This article	n/a
**Software and algorithms**		
PRISM	GraphPad	Version 9
SnapGene	SnapGene.com	Version 4.2.11
BioRender	Biorender.com	Biorender.com
sgRNA designer	Broad Institute	CRISPick
RNA folding designer	urmc.rochester.edu/rna	Matthews Lab RNA structure
Celigo Imaging Software	Nexcelom	Version 5.5
ICE CRISPR analysis tool	Synthego	ICE version 2
Image Lab	Bio-Rad	Version 6.1
MinKNOW	Oxford Nanopore Technologies	Version 21.06
CFX Maestro Software	Bio-Rad	**#12013758**

*N/A, non-applicable.

### Cell lines

Vero cells [African green monkey (*Chlorocebus aethiops*), kidney], BSR-T7 cells [golden hamster *(Mesocricetus auratus*), kidney] and U373-MG gH/gL/UL128 cells (*Homo sapiens*, astrocytoma) were cultured in Dulbecco’s Modified Eagle’s Medium (DMEM) supplemented with 10% heat-inactivated FBS. MRC-5 cells (Homo sapiens, foetal lung fibroblasts) were cultured in Eagle’s Minimum Essential Medium supplemented with 10% heat-inactivated FBS or in Lebovitz’s L-15 media supplemented with 10% heat-inactivated FBS. ARPE-19 cells (Homo sapiens, retinal epithelial cells) were cultured in DMEM and F-12 media in a 1:1 ratio with 10% heat-inactivated FBS. HUVEC cells (Homo sapiens, endothelial cells) were cultured in EGM-2 Bullet Kit media with 10% heat-inactivated FBS. All cells were tested monthly for mycoplasma contamination (Lonza, MycoAlert Plus Myco detection kit). Cells were cultured at 37 °C in a humidified atmosphere (5% CO_2_). MRC-5 cells cultured in a closed roller bottle system were kept in a 37 °C warm room in L-15 media.

### Viruses

HCMV strain TB40/E and HCMV strain AD169 were prepared by infecting MRC-5 cells with HCMV in a roller bottle at an m.o.i. of 0.01 and culturing for 10–20 days at 37 °C, until extensive cytopathic effect (CPE) was evident. HCMV concentrated stock was prepared by freeze-thawing the infected cells and spinning out cellular debris, layering the supernatant over a 20% sorbitol cushion and pelleting the virus at 70,000 ***g*** for 90 min prior to resuspending the viral pellet in 3% BSA in H_2_O. Cell-free virus stock was prepared by pelleting only the cleared supernatant from infected cells.

Our SeV-CRISPR/Cas vector is derived from the trypsin-independent F1R strain. The F1R strain contains six mutations in the F protein (compared to the parental Z strain) that render it trypsin-independent [25,27]. SeV is not pathogenic in humans and has been used in gene therapy trials as well as a Jennerian vaccine in infants and children [15,28, 29]. The safety of our SeV-CRISPR/Cas system is further enhanced by multiple mutations engineered to confer temperature sensitivity at physiological temperatures (37 °C) [20]. All recombinant virus work has been approved by our Institutional Biosafety Committee under protocol SPROTO202400000157.

Recombinant *ts* SeV-Cas9 viruses were rescued according to an optimized reverse genetic protocol previously described [18]. Briefly, BSR-T7 cells were seeded into 6-well plates and transfected with a T7-opt plasmid (pCAGGS), SeV helper plasmids under the T7 promoter (for SeV N, P and L) and the SeV-Cas9 T7-driven, anti-genomic viral plasmid using Lipofectamine-LTX (#15338100). Cells were placed at 32 °C and monitored for rescue, evidenced by the expression of GFP from the reporter virus. The supernatant from viral rescue cells (passage 0) was used to infect BSR-T7 cells in T-175 flasks. When 90% of the cells were GFP+, DMEM was replaced with Opti-MEM supplemented with 2% FBS and penicillin-streptomycin, and the cells were incubated for 24 h at 32 °C. Viral supernatant was cleared, and SeV-Cas9 virus was concentrated through sucrose layering (65% and 20% sucrose, respectively) and ultracentrifugation at 100,000 ***g*** for 2 h. The concentrated virus was removed from the tubes via a syringe. All SeV-Cas9 virus stocks were produced in cells cultured at 32 °C for this study given the *ts* nature of the SeV-Cas9 virus.

### Method details

#### Cloning of recombinant SeV-Cas9 viruses and plasmids

All recombinant SeV-Cas9 viruses were cloned using the parental *ts* SeV-Cas9 backbone described in Stevens *et al*. [20] (GenBank: PV665661). gRNAs targeting the HCMV genes UL128, UL130 and US2 were designed using the Broad Institute sgRNA designer. gRNAs that were predicted to fold correctly between the flanked ribozymes in the gRNA cloning cassette (using the RNAstructure webserver tool [30]) were created with overlap PCR (CloneAmp HiFi PCR mix) and cloned into the parental *ts* SeV-Cas9 backbone (InFusion HD) between the P and M genes. Unique restriction sites between the P and M genes were utilized to facilitate cloning. An annotated SnapGene file of SeV-CRISPR Cas ts_UL128 g1 encoding the UL128 g1 cassette is attached as Supplementary Data for reference. Full-length SeV-Cas9 viral genomes were maintained in Stbl2 *Escherichia coli* (ThermoFisher) grown at 30 °C. A 500 bp region of the HCMV gene UL83 was cloned into the pGEM-T easy vector with TA cloning (Promega), and the plasmid was maintained in Δmrr-hsdRMS-mcrBC and ΔmcrA Stellar™ competent cells (TaKaRa). All primers used in cloning are listed in Table S1.

#### Viral titration

All *ts* SeV-Cas9 viruses used in this project contain an EGFP reporter. To titrate *ts* SeV-Cas9, viral stocks were serially diluted and added to Vero cells in a 96-well plate. Cells were incubated at 32 °C for 36–48 h post-infection, and plates were scanned on a Celigo Image Cytometer (Nexcelom). The number of GFP+ cells was counted, and the viral titre was calculated as IU per millilitre (IU ml^−1^).

To determine the titre for HCMV, viral stocks were serially diluted and added to either MRC-5 or ARPE-19 cells in a 96-well plate and incubated at 37 °C. At 24 hpi, cells were fixed with 4% paraformaldehyde, permeabilized with 0.3% Triton X-100 and blocked with 5% normal goat serum for 30 min. Cells were stained with primary IE1/IE2 rabbit polyclonal antibody (1:1,000 dilution) for 1 h, rinsed 3×, were stained with secondary goat anti-rabbit Alexa 488 antibody (1:1,000 dilution), rinsed 3× and were stained with DAPI. Plates were scanned on a Celigo Image Cytometer (Nexcelom) and the number of IE1-positive cells was counted, and the viral titre was calculated as IU mL^−1^.

#### Co-infection method with SeV-Cas9 and HCMV

For all SeV-Cas9 editing experiments, cells were infected with *ts* SeV-Cas9 at an m.o.i. of 10 for 1 h at 32 °C, the viral inoculum was removed and cells were incubated at either 32 °C (MRC-5 and ARPE-19 cells) or 34 °C (HUVECs) for 2 days. Viral infection with SeV-Cas9 was confirmed by scanning the cells with the Celigo Image Cytometer and verifying all cells were green fluorescent protein positive. Next, cells were infected with HCMV TB40/E at an m.o.i. of 0.1 and incubated at 37 °C. End-point samples were collected by harvesting the cells and supernatant together and stored at −80 °C. For dual editing experiments, MRC-5 cells were infected with equal amounts of SeV-Cas9 UL128 and SeV-Cas9 UL130 (each at an m.o.i. of 5) for 2 days at 32 °C prior to infection with HCMV TB40/E.

#### Viral DNA extraction and analysis

To extract viral DNA (vDNA), infected cells were scraped into the media and the mixture went through one freeze-thaw cycle to release cell-associated virus. Cellular debris was cleared via brief centrifugation, and 200 µl of supernatant was processed with the PureLink vDNA kit (ThermoFisher). PCR was used to amplify the section of the HCMV genome targeted for CRISPR editing (i.e. UL128, UL130 or US2) with the extracted vDNA serving as the template. PCR products were purified and sent for sequencing analysis (Macrogen), and Sanger sequencing files were analysed for CRISPR editing efficiency with ICE (Synthego).

#### Plaque purification of edited HCMV

To isolate clones of edited HCMV, the supernatant containing pooled edited HCMV was serially diluted, used to infect MRC-5 cells in a 12-well plate and overlaid with a low melting temperature agarose. Infected cells were incubated for 10–20 days post-infection, until plaques were visible. Using a 20 µl filter tip, individual plaques were picked and used to infect MRC-5 cells in a 24-well plate. These cells were incubated at 37 °C until visible CPE was present throughout the well. Cells and supernatant were then harvested and processed for vDNA extraction and sequencing, to confirm the edited virus was pure (i.e. only one INDEL present per plaque picked). Verified clonal isolates of HCMV with edited UL128 and UL130 were grown as viral stocks.

#### Immunoprecipitate and immunoblotting

MRC-5 cells were infected with HCMV TB40/E or clonal isolates of HCMV with edited UL128 and UL130 at an m.o.i. of 1. When CPE was highly evident (between 6 and 11 dpi), cells were rinsed 1× in PBS, scraped off the plate, aliquoted to microfuge tubes and snap-frozen prior to storage at −80 °C. Cells were then lysed in NP-40 buffer with protease inhibitors for 30 min on ice and cleared of cellular debris, and protein concentration was calculated by the Bradford assay. Cell lysates from uninfected U373-MG gH/gL/UL128 cells were used as a positive control for the immunoprecipitate (IP). For the IP, 5 µg of gH mAb 11D3 was added to each sample, which were rotated at 4 °C for 1 h, after which protein A/G beads were added to each tube and samples were rotated overnight at 4 °C. Beads were then rinsed 3× in FLAG buffer, and samples were eluted by adding 1× sample buffer with DTT and boiling for 5 min.

For immunoblots, samples were run on pre-cast 4–15% SDS mini gels (Mini-PROTEAN Tetra cell), transferred to PVDF membranes (TurboBlot) and incubated with Intercept blocking buffer (Li-Cor) for 30 min. Membranes were incubated with primary antibodies for either 1 h at room temperature or at 4 °C overnight. The UL128 rabbit antibody was used at a 1:500 dilution, the gL rabbit antibody was used at a 1:1,000 dilution and Cytogam was used at a 1:2,000 dilution. Membranes were washed 3× in phosphate bufferred saline with tween (PBST), incubated with secondary antibodies for 1 h at RT and washed 3× in PBST. Goat anti-rabbit A647 (ThermoFisher) was used at 1:1,000, and Donkey anti-HRP (Southern Biotech) was used at 1:10,000. Blots were imaged on the Bio-Rad ChemiDoc or were processed for film development.

#### ONT sequencing

HCMV vDNA was extracted from co-infected MRC-5 cells at 8 dpi. Primers flanking the region of the HCMV genome encoding UL128 and UL130 were used to create a 3.4 kbp amplicon using a Q5 HiFi DNA polymerase (NEB). The amplicons were PCR-purified, processed with formalin-fixed paraffin-embedded DNA repair and end repair/dA-tailing (NEBNext Companion Module) and quantified with the Qubit™ dsDNA BR assay (ThermoFisher). Amplicons were next barcoded following the ONT native barcode ligation protocol (EXP-NBD104), adapters were added (NEBNext Companion Module), the prepared DNA library was added to the primed SpotON Minion Flow cell (R9 version) and sequences were acquired for 36 h using the MinKNOW software interface. A custom Python script was developed to identify deletions and insertions from the alignment of sequencing reads in a sequence alignment map file. The script processes these alignments and identifies the proportion of reads with deletions or insertions based on user-defined criteria (such as minimum mutation size), which are output as a comma separated values file.

#### Quantitative polymerase chain reaction (qPCR)

To quantify HCMV genome copy number in viral samples, 200 µl of each sample was first treated with DNase I for 10 min to degrade any DNA not protected by viral capsids and then inactivated by 25 mM EDTA. vDNA was extracted using the PureLink viral DNA kit (ThermoFisher) and was eluted in 50 µl of RNase-free water. A highly conserved region of the UL83 gene was used as the amplicon to quantify genome copies using the Sensi Fast SYBR and Fluorescein kit (Bioline) (primers listed in Table S1). PCR reactions were assembled in a volume of 20 µl, containing 200 nM of forward and reverse primers, 10 µl of the fluorescein MM and 5 µl of diluted vDNA. Each sample was measured in duplicate. The PCR products were detected using the CFX96 Touch Real-Time PCR detection system (Bio-Rad). Serial dilutions of the pGEM-T UL83 standard plasmid were used to create a standard curve, and the qPCR results for HCMV were calculated as viral genomes per millilitre.

#### Quantification and statistical analysis

Viral growth curve, SeV-Cas9 editing experiments in MRC-5 cells and qPCR experiments were all performed in three independent replicates. The editing experiments in ARPE-19 and HUVECs and the titration of isogenic HCMV clones were done in two separate biological experiments in technical duplicates. The immunoprecipitations and western blotting were performed three times, with the clearest images being chosen for this paper. The dual-editing experiment for ONT analysis was performed three times, and amplicons from two of the experiments were processed, barcoded and run together on the same minION flow cell.

#### Reagent availability

All plasmids for generating the recombinant SeV-CRISPR/Cas system are available from the corresponding authors upon request and subject to a material transfer agreement from Mount Sinai Innovation Partners.

## Results

### Devising a co-infection strategy to edit HCMV with a SeV-Cas9 viral vector

**Fig. 1.**
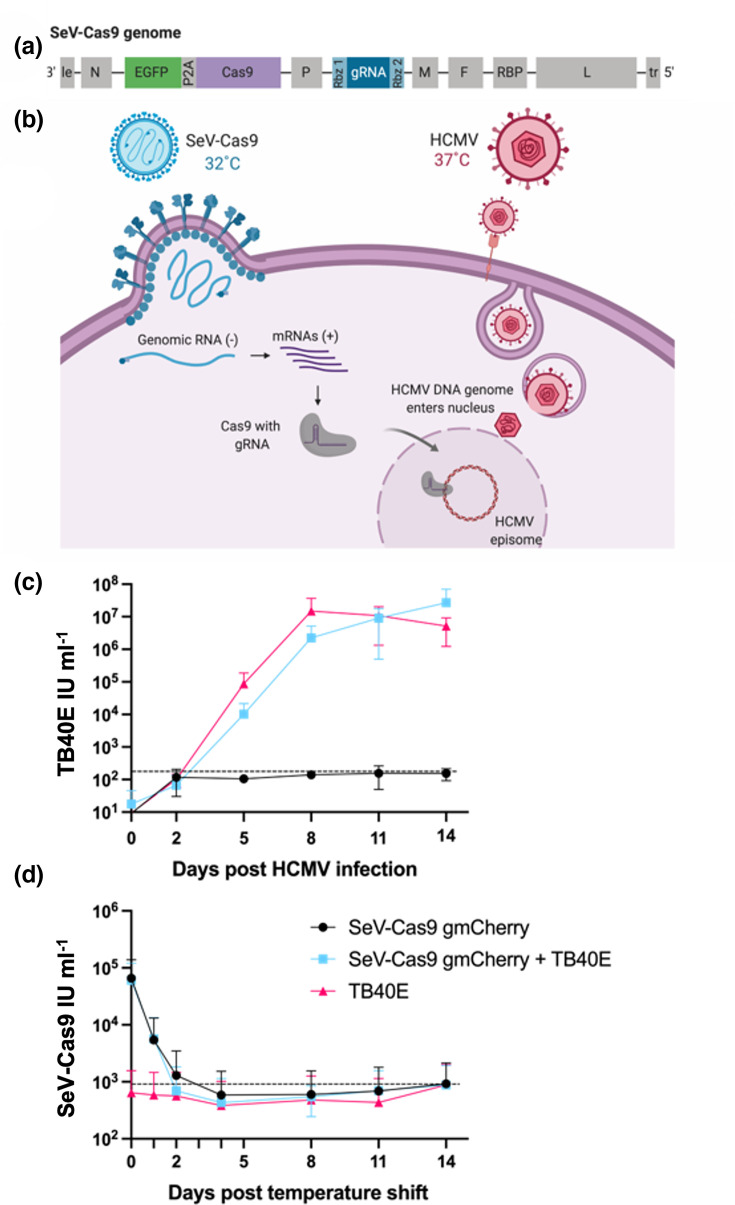
SeV-Cas9 and HCMVg co-infection strategy. (**a**) A depiction of the *ts* SeV-Cas9 viral genome containing the SeV genes (light grey), the EGFP reporter (green) and Cas9 (dark grey) separated by a P2A linker and the gRNA cassette (dark blue) flanked by two *cis*-acting ribozymes (light blue). Rbz1 is a hammerhead ribozyme, while Rbz2 is an hepatitis D virus (HDV) ribozyme. (**b**) The co-infection strategy to deliver Cas9 and gRNA with SeV begins with infecting cells with *ts* SeV-Cas9 and incubating the cells for 2 days at the permissive temperature of 32 ˚C, allowing for viral transcription and the production of Cas9 and gRNA, which is delivered to the nucleus. At 2 dpi with SeV-Cas9, the cells are infected with HCMV and then incubated at 37 ˚C, allowing for HCMV viral entry and replication and blocking further *ts* SeV-Cas9 replication. After the HCMV genome enters the nucleus, the Cas9/gRNA complex targets the HCMV genome for editing. (**c**) MRC5 fibroblasts were infected with *ts* SeV-Cas9 gmCherry at an m.o.i. of 10 and incubated for 2 days at 32 ˚C prior to infection with HCMV strain TB40/E at an m.o.i. of 0.1. At 2, 5, 8, 11 and 14 dpi with HCMV, samples were collected and SeV-Cas9 and HCMV viral titres were determined. This was done in three independent replicates. (**d**) The supernatant harvested in (c) was also measured for infectious SeV-Cas9, and titres were calculated. The dashed lines in (c) and (d) indicate limits of detection.

A major benefit of the SeV-Cas9 viral vector system is that it allows for an all-in-one delivery of Cas9 and gRNA. SeV, like all paramyxoviruses, has six canonical transcriptional units (N, P, M, F, RBP and L) expressing the six genes essential for viral replication (Fig. 1a). N (nucleoprotein), P (phosphoprotein) and L (large protein comprising the RNA-dependent RNA polymerase) form the replicase complex, whereas the M (matrix), F (fusion) and RBP (receptor-binding protein) are the structural proteins required for viral assembly, budding and entry. RBP replaces the old nomenclature for paramyxovirus attachment glycoprotein, which was variously termed HN, H or G [31]. Each viral gene comprises a 5′ and 3′ UTR that flank the cognate ORF. The viral transcriptase recognizes specific gene start and gene stop sequences in these UTRs and generates a capped polyadenylated mRNA encoding each ORF. In our SeV-Cas9 vector, the viral genome encodes two additional transcriptional units: an EGFP-P2A-Cas9 expression cassette between the N and P genes and the gRNA expression module between the P and M genes (Fig. 1a). The gRNA is expressed embedded as part of a capped and polyadenylated ‘mRNA’ with 5′ and 3′ UTRs derived from the virus. The extraneous virus-derived sequences are cleaved by two *cis*-acting ribozymes to generate the authentic gRNA that is competent for loading into the Cas9 (Fig. 1a, Rbz1 and Rbz2). Viral rescue of this cDNA genome with reverse genetics produces a replication-competent SeV-Cas9 virus capable of delivering Cas9 and gRNA upon infection [32]. When targeting a host gene with CRISPR/Cas9, the recombinant SeV-Cas9 is used to infect the cells of interest, and the Cas9/gRNA produced during infection will edit the host genome. But to target the HCMV genome for editing with SeV-Cas9, a co-infection method must be utilized. Our *ts* SeV vector has the added benefit of being IFN-silent [20], which is also essential for our co-infection method.

For this approach, cells were initially infected with the SeV-Cas9 virus containing a gRNA targeting the HCMV gene of interest (Fig. 1b). Infection with a high m.o.i. of 10 ensures that all cells will produce the Cas9/gRNA complex. Since the SeV-Cas9 viral vector contains mutations rendering it ts, infected cells are incubated at the permissive temperature of 32 °C. During this period, the transcription of the viral genome produces Cas9 and the gRNA. The gRNA/Cas9 complex is then transported to the nucleus to edit its target DNA. After 2 days of SeV-Cas9 infection at 32 °C, cells are then co-infected with WT HCMV at an m.o.i. of 0.1 and transferred to 37 °C. This low m.o.i. ensures that infected cells will only receive one HCMV virion. Furthermore, incubation of these cells at 37 °C halts SeV-Cas9 replication [20], while HCMV proceeds through its replication cycle, resulting in delivery of the viral genome to the nucleus, where it circularizes and forms an episome. At this point, the SeV-delivered Cas9/gRNA complex can presumably target the HCMV genome for editing. If the incoming HCMV genome is successfully edited before viral genome replication begins, then newly produced virions will contain edited genomes (Fig. 1b).

Before conducting an editing experiment, we first needed to demonstrate that HCMV could enter and replicate in cells previously infected with SeV-Cas9. To test this, fibroblasts were infected with a SeV-Cas9 vector carrying a gRNA targeting mCherry, which produces a functional Cas9/gRNA complex but will not target or edit either host or HCMV viral DNA. After 2 days of incubation at 32 °C, cells were infected with WT HCMV strain TB40/E and shifted to 37 °C. We found that prior infection with SeV-Cas9 did not limit HCMV replication, as viral growth curves for HCMV were not altered by SeV-Cas9 co-infection (Fig. 1c). In addition, no detectable SeV-Cas9 was present after day 4 of the temperature shift to 37 °C, confirming that SeV-Cas9 is *ts* (Fig. 1d). These data suggest that a co-infection strategy to edit HCMV using SeV-Cas9 may be possible and demonstrate that HCMV samples harvested from SeV-Cas9 co-infected cells will not contain infectious SeV-Cas9 if harvested 4 or more days post-temperature shift.

### Targeting the HCMV PC with SeV-Cas9

**Fig. 2.**
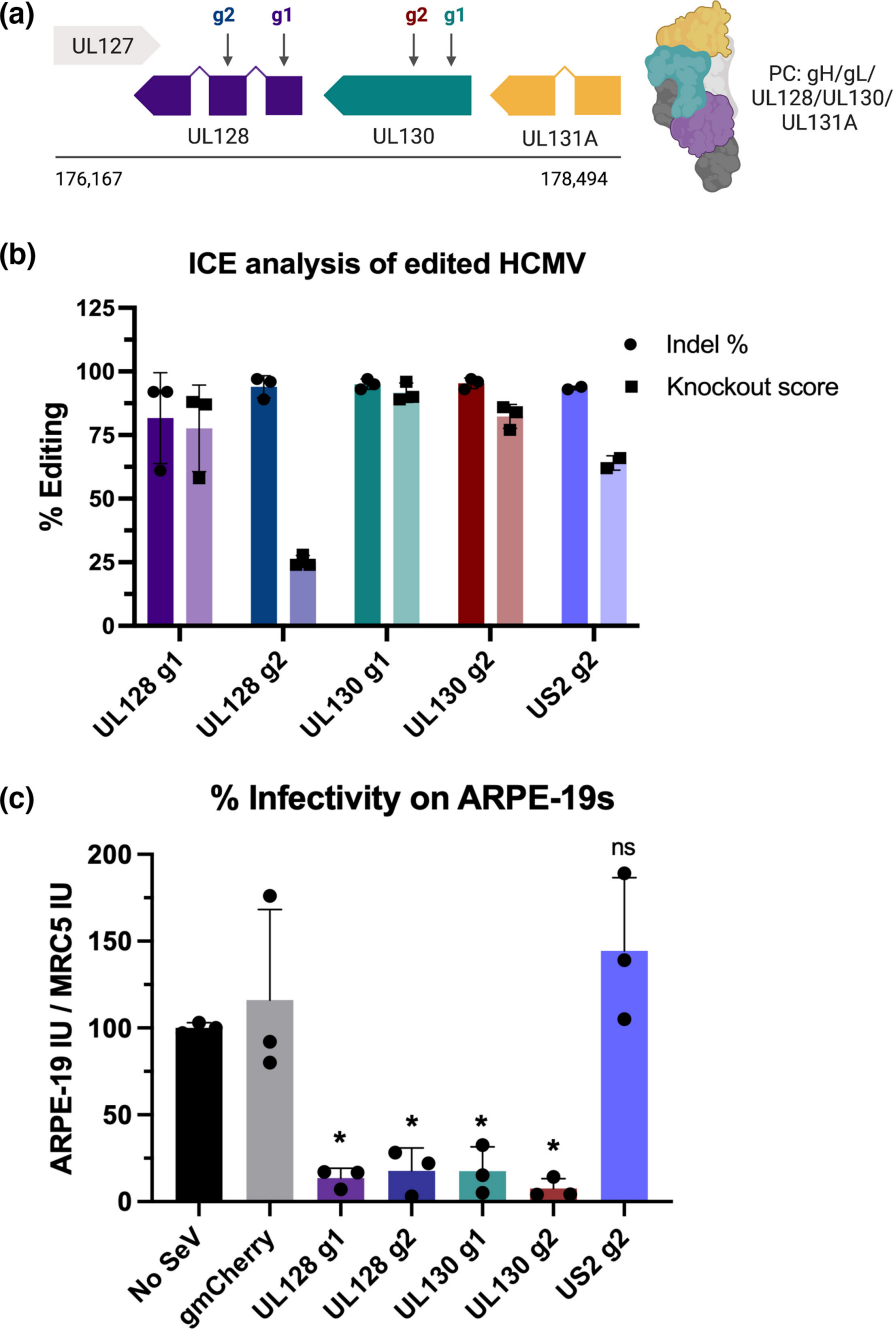
SeV-Cas9 targets the HCMV PC with high efficiency. (**a**) A schematic of the UL127-UL131A gene block in HCMV with the estimated location of the designed gRNAs targeting UL128 and UL130 shown as g1 and g2. (**b**) Viral DNA from HCMV-edited virions was extracted, sequenced and analysed for CRISPR editing efficiency using ICE analysis. The percentage of indels (insertions or deletions) and knockout scores were calculated for three independent experiments. (**c**) Edited HCMV virions were titrated on MRC5 fibroblasts and ARPE-19 epithelial cells. The per cent infectivity of each sample was normalized to the non-edited HCMV control. Data are from three independent experiments, and statistical significance was calculated using an unpaired t-test comparing each sample to the No-SeV control. Statistical significance is denoted as *P<0.01.

The broad cell tropism of HCMV *in vivo* relies, in part, on the PC comprised of gH/gL/UL128/UL130/UL131a (Fig. 2a). To gain entry into epithelial cells, endothelial cells and monocytes, the virus requires an intact PC to bind the various receptors and entry factors on these cell types [33,36]. When the PC is disrupted by a mutation occurring in UL128, UL130 or UL131A, viral entry is restricted to infection of only fibroblasts [9,37, 38]. Viral strains lacking the PC can only facilitate entry using the trimer (composed of gH/gL/gO), and fibroblasts are the only cell type that support trimer-mediated HCMV entry [39]. In short, HCMV with a functional PC can enter all relevant cell types, while HCMV lacking the PC can only enter fibroblasts.

Since the role of the PC during viral entry is well documented, we targeted the HCMV PC with SeV-Cas9 as a proof-of-principle experiment. We designed two gRNAs to target UL128 and two gRNAs to target UL130, cloned them into the SeV-Cas9 vector and rescued these viruses (Fig. 2a). Using the co-infection strategy depicted in Fig. 1(b), we infected fibroblasts with the SeV-Cas9 UL128 g1 virus followed by HCMV strain TB40/E, which contains an intact PC [39]. Viral HCMV DNA harvested at 4, 6 and 8 dpi showed cognate indels (insertions and deletions) in UL128 that increased from slightly more than 50% (4 dpi) to 75% (6 dpi) and 90% (8 dpi), respectively (Fig. S1A, available in the online Supplementary Material). Samples harvested from co-infected cells increased in HCMV titre over time that was comparable to a no SeV control (Fig. S1B). This indicates that SeV-Cas9 editing of the HCMV genome did not prevent HCMV from continuing through its replication cycle and producing infectious particles. Furthermore, the UL-128 bulk-edited viruses were severely impaired in their ability to infect epithelial (ARPE-19) cells (Fig. S1C). Given these results, we selected 8 days post-infection as the harvest time point for all future editing experiments.

Following the co-infection strategy, we found that each of the SeV-Cas9 viruses targeting UL128 and UL130 induced high levels of HCMV editing, with the total per cent editing reaching 90% in most samples (Fig. 2b). To determine if SeV-Cas9 editing of HCMV disrupts the protein production of UL128 and UL130, thus blocking the formation of an intact PC, we titrated the virus on fibroblasts and epithelial cells. If the PC has been successfully disrupted, then the edited HCMV TB40/E should lose the ability to infect ARPE-19 epithelial cells. Notably, each of the UL128 and UL130 edited viruses was significantly reduced in their ability to infect epithelial cells (Fig. 2c). To verify it was UL128 and UL130-specific editing that prevented epithelial infection and not an off-target effect of SeV-Cas9 editing of the HCMV genome, we designed and rescued a SeV-Cas9 virus with a gRNA targeting the US2 gene. US2 is an HCMV protein that downregulates MHC class I to subvert the host immune response, and it has no known role in viral tropism [40,41]. SeV-Cas9 targeting US2 exhibited a 90% editing efficiency and US2-edited HCMV infected epithelial cells with no defect (Fig. 2b, c). Thus, the loss of infectivity on epithelial cells for the SeV-Cas9 UL128 and UL130-edited HCMV was specifically due to the editing of the components of the PC genes.

### Isolating isogenic clones from SeV-Cas9-edited HCMV

Indels are formed when the DNA double-stranded breaks induced by CRISPR/Cas9 are fixed by the endogenous DNA repair machinery and nt are either added or deleted in the process [42]. Many indels will cause a disruption of the coding sequence, resulting in premature stop codons and the production of non-functional proteins. In each CRISPR/Cas9 experiment, the edited DNA will exist as a group of different indels pooled together. When the editing efficiency is high, the likelihood of gene knockout is also high. This phenomenon is illustrated by the SeV-Cas9 editing of the PC. The 90% indel rate in UL128 and UL130-edited HCMV virions is reflected in the 90% reduction in epithelial cell infectivity (Fig. 2b, c). However, there is not a complete knockout of PC activity because an estimated 10% of HCMV virions remain unedited and not all indels will result in a gene knockout.

**Fig. 3.**
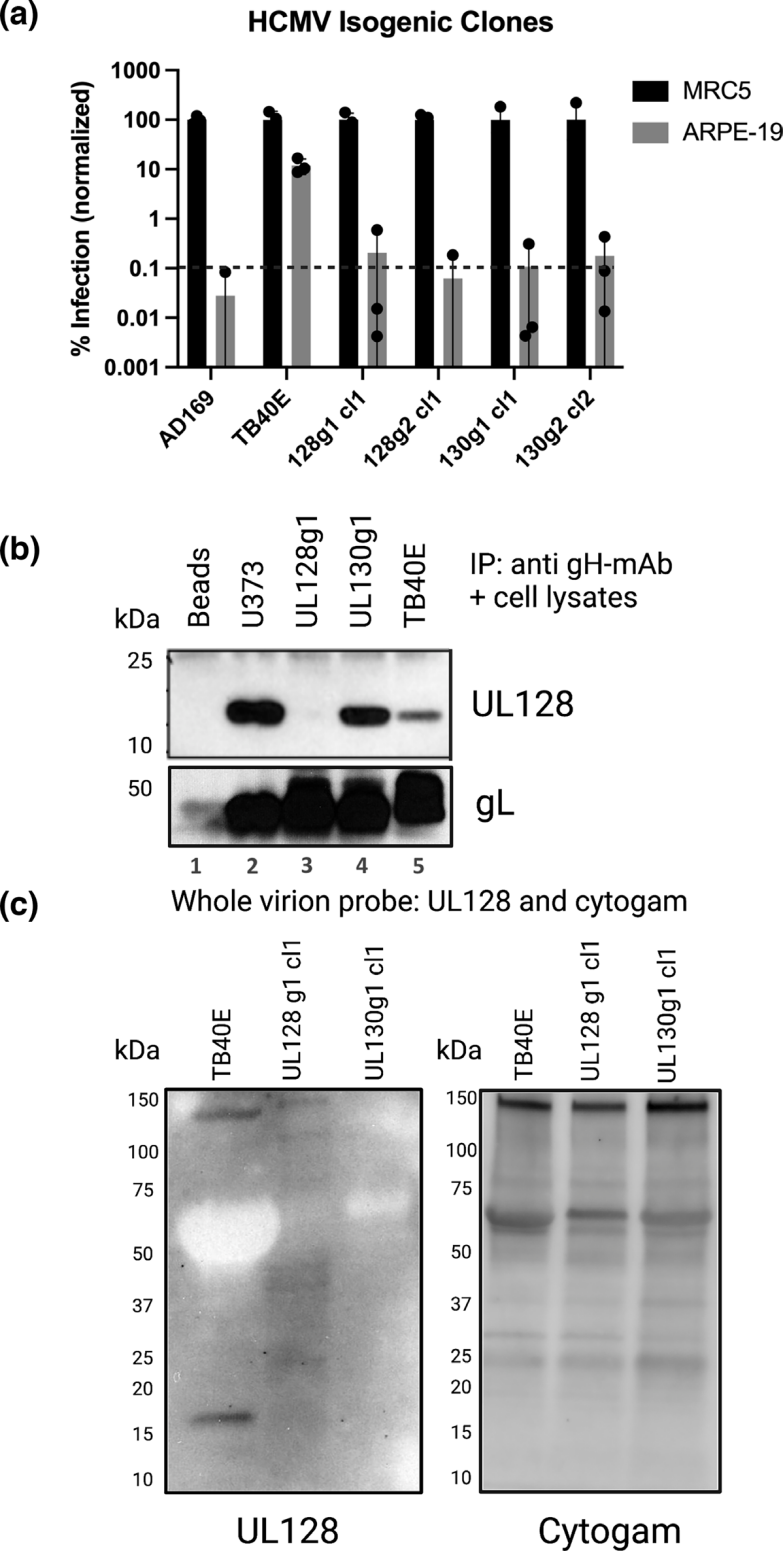
SeV-Cas9 editing allows for the isolation of isogenic HCMV clones. (**a**) HCMV virus was titrated onto MRC5 fibroblasts and ARPE-19 epithelial cells, and the normalized infection was calculated by comparing viral infectivity on ARPE-19 cells to MRC5 cells. The strain AD169 served as a negative control, TB40/E was the positive control and the isogenic, SeV-Cas9-edited HCMVs were the experimental samples. This experiment was performed in triplicate, and statistical significance was determined using a two-way ANOVA with Sidak’s multiple comparison test. (**b**) PC from infected cell lysate was immunoprecipitated using a gH mAb and run under reducing conditions on SDS-PAGE. Blots were probed with antibodies against UL128 and gL. (**c**) Concentrated HMCM viral stock (1E+07 per sample) was run under reducing conditions on SDS-PAGE. Blots were probed with anti-UL128 antibodies, to probe for the PC, or Cytogam to determine viral loading levels and PP65 content in the virions. This experiment was repeated three times.

To isolate clonal knockouts, we plaque purified the edited HCMV samples harvested from SeV-Cas9-infected cells. Individual plaques were picked and expanded in fibroblasts, and the viral DNA was sequenced for each sample. We found that, of the plaques that successfully expanded, between 50 and 67% of the plaques were clonal knockouts containing a predicted truncation due to a premature stop codon (Table 2). In a single round of plaque purification, we isolated isogenic clones for HCMV containing UL128 or UL130 knockouts. While the pooled edited HCMV showed a 1 log reduction in epithelial infectivity, the isogenic PC-edited HCMV is reduced by 3 logs (Figs2c  3a). Furthermore, these clones exhibited similar levels of infectivity on epithelial cells compared to the AD169 lab strain of HCMV, which lacks a functional PC (Fig. 3a). Given that the isogenic HCMV clones behave like AD169, the PC is fully disrupted in the PC-edited viral clones.

**Table 2.** Plaque-purified isogenic HCMV clones. Pooled edited HCMV virus for each PC-targeting guide was plaque-purified. The percentages of plaques that expanded, were isogenic and contained a predicted knockout are listed along with the predicted disruption induced by indel formation

Sample	Plaque picked	Plaque expanded	Clone with predicted knockout, *N* (%)	Sample	Edit	Isolation	Predicted disruption
UL128 g1-edited CMV	10	8	4 (50)	UL128 g1 P1	+1 indel	**Yes**	Stop codon at AA 71
				UL128 g1 P2	Mixed	No	
				UL128 g1 P3	−1 indel	**Yes**	Stop codon at AA 33
				UL128 g1 P4	Mixed	No	
				UL128 g1 P5	+1 indel	**Yes**	Stop codon at AA 71
				UL128 g1 P6	Mixed	No	
				UL128 g1 P7	Mixed	No	
				UL128 g1 P8	−7 indel	**Yes**	Stop codon at AA 33
UL128 g2-edited CMV	10	5	3 (60)	UL128 g2 P1	−1 indel	**Yes**	Stop codon at AA 81
				UL128 g2 P2	−1 indel	**Yes**	Stop codon at AA 81
				UL128 g2 P3	−1 indel	**Yes**	Stop codon at AA 81
				UL128 g2 P4	Mixed	No	
				UL128 g2 P5	Mixed	No	
UL130 g1-edited CMV	10	3	2 (67)	UL130 g1 P1	−1 indel	**Yes**	Stop codon at AA 30
				UL130 g1 P2	Mixed	No	
				UL130 g1 P3	+1 indel	**Yes**	Stop codon at AA 43
UL130 g2-edited CMV	10	3	2* (67)	UL130 g2 P1	−6 indel	No	In-frame deletion
				UL130 g2 P2	−4 indel	**Yes**	Stop codon at AA 77
				UL130 g2 P3	−1 indel	**Yes**	Stop codon at AA 77

* An additional clone contained a 6 bp in-frame deletion which may or may not be functional.

### The PC is knocked out in SeV-Cas9-edited HCMV clones

Since we isolated predicted clonal knockouts of UL128 and UL130 in edited HCMV, we wanted to verify that these viruses were truly lacking a PC. In cells infected with TB40/E, it is possible to IP the PC using an anti-gH antibody and probe for the presence of PC components. To test this, we first used cell lysate from U373 cells stably expressing gH, gL and UL128; these proteins form a covalent complex and have been used as a proxy for the PC in other studies [43]. The anti-gH antibody was able to IP the gH/gL/UL128 complex from U373 cells as both UL128 and gL were detected with an immunoblot (Fig. 3b, lane 2). In cell lysate from TB40/E, UL128g1 isogenic HCMV and UL130g1 isogenic HCMV-infected cells, gL was detected in all samples (Fig. 3b, lanes 3–5). This is expected since gH and gL form a disulphide-linked heterodimer as part of the trimer and PCs, and their interaction is not disrupted when UL128 or UL130 are absent [23,44]. As expected, UL128 is able to IP with gH from TB40/E-infected cell lysate, indicating an intact PC. Conversely, there is no detectable UL128 in the UL128g1-edited infected cell lysate, which is also expected because the isogenic UL128g1 HCMV clone contains a premature stop codon. Finally, UL128 is present in the UL130g1-edited infected cell lysate, as the absence of UL130 does not prevent UL128 from covalently interacting with gH/gL (Fig. 3b) [23].

Next, we probed whole edited virions for the PC. If even one of the PC components is absent, an intact PC will not form on the surface of the virion. Purified cell-free virions of TB40/E, UL128g1 isogenic HCMV and UL130g1 isogenic HCMV were resolved on an SDS-PAGE gel under reducing conditions. Only virions from TB40/E had detectable UL128 (15–17 kDa); both isogenic knockouts of UL128 and UL130 did not package UL128 into the virion (Fig. 3c). Equal amounts of virions were loaded for each sample as evidenced by the pp65 levels observed by probing the blots with Cytogam (Cytomegalovirus Immune Globulin). These data biochemically confirm that SeV-Cas9 editing of HCMV can functionally knock out UL128 and UL130.

### Precision knockout of the PC with a dual editing strategy

**Fig. 4.**
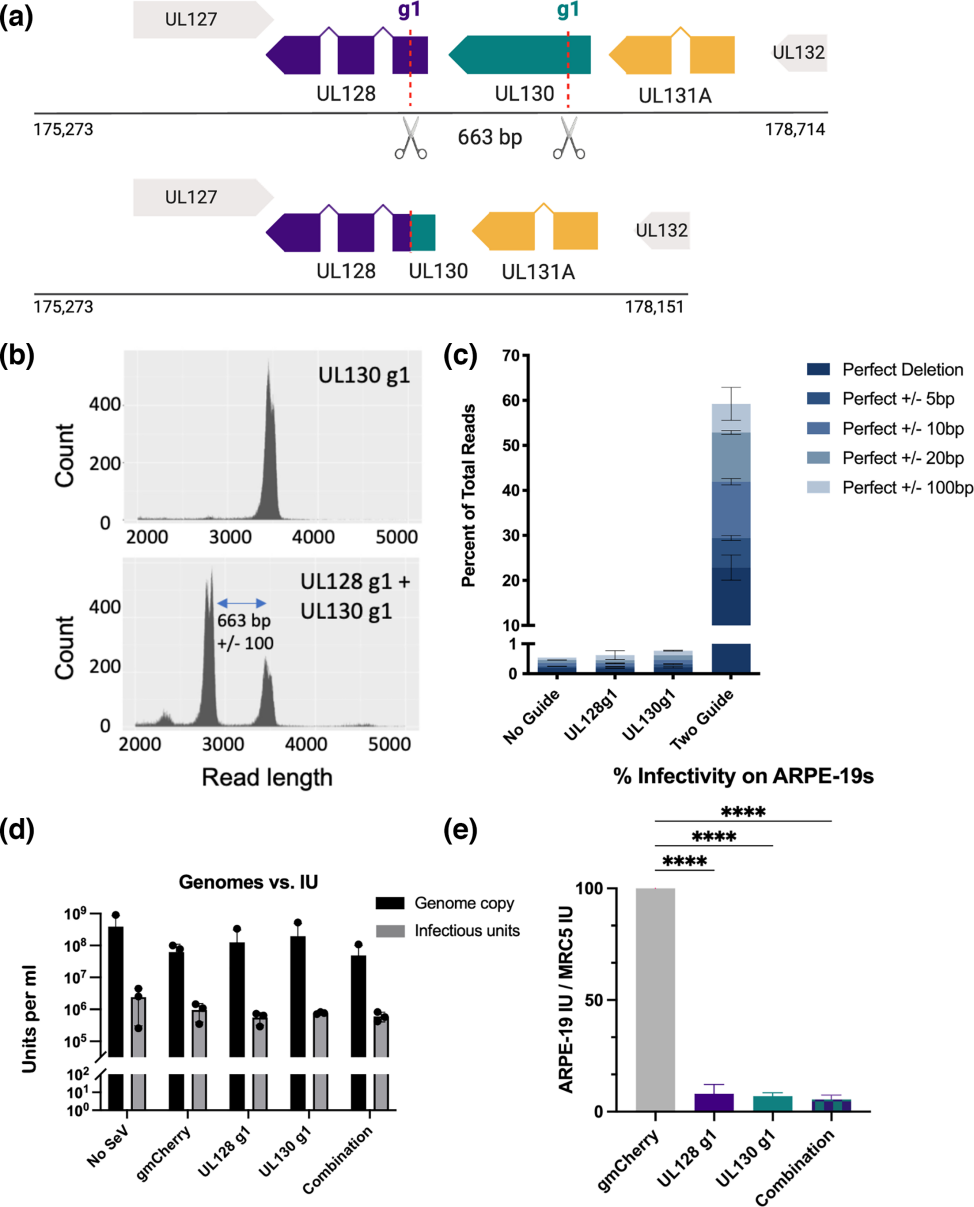
Dual editing of the HCMV genome with two SeV-Cas9 viruses. (**a**) A schematic of the HCMV pentamer gene block and the relative location of the UL128 g1 and UL130 g1 cut sites. The bottom image depicts the 663 bp deletion that is predicted to occur if SeV-Cas9 UL128g1 and SeV-Cas9 UL130 were employed in the same editing experiment. (**b**) Long-read ONT amplicon sequencing of HCMV edited by one (UL130g1, top panel) or two (UL128g1+UL130 g1, bottom panel) SeV-Cas9 targeting the PC. The read distributions are shown. Data from these reads are plotted in (**c**). Viral DNA from HCMV virions edited either by one PC targeting SeV-Cas9 or by two guides was extracted and analysed by ONT sequencing. The percentages of total reads containing the predicted perfect deletion are mapped for each sample. This experiment was repeated in two biological replicates. (**d**) HCMV virus edited by the single PC-targeting SeV-Cas9 or by the combination was titrated on MRC5 fibroblasts to determine the infectious unit per millilitre, and viral DNA was extracted and genome copy number per millilitre was calculated by quantitative PCR (qPCR). The genome copy versus infectious units was plotted from three independent replicates, and the differences were not statistically significant, as determined by multiple unpaired t-tests. (**e**) UL128g1, UL130g1 or combination edited HCMV virus was titrated on MRC5s and ARPE-19s, and the per cent infectivity was calculated. Statistics were calculated using a one-way ANOVA test with *P*<0.0001.

Multiplexing approaches are commonly used to increase CRISPR editing efficiency [45]. To determine if this method would increase SeV-Cas9 editing of HCMV, we performed a dual editing experiment. We infected cells with equal amounts (each at an m.o.i. of 5) of SeV-Cas9 UL128g1 and SeV-Cas9 UL130g1 to deliver both gRNAs prior to infecting the cells with HCMV. Based on the predicted cut sites for the gRNAs, if UL128g1 and UL130g1 target the same HCMV genome, a section of 663 bp of the genome will be removed. Assuming the HCMV genome can re-ligate, the majority of UL130 and part of UL128 will be deleted from the virus (Fig. 4a). Since large deletions cannot be detected with traditional sequencing methods, we used ONT long-read sequencing for amplicon analysis of a 3.4 kb fragment surrounding the Cas9/CRISPR cut sites in UL128 and UL130. No large deletions in the PC region were detected when only SeV-Cas9 UL130g1 was used, as indicated by the distribution of read counts around the predicted amplicon size (Fig. 4b, top). However, when the two guides were used together, we found that more than 60% of the viral DNA reads contained large deletions in the PC region, with the majority of the deletions resulting in the expected 663 (±100 bp) deletion (Fig. 4b, bottom, Fig. 4c).

One potential concern is that deleting a section of the HCMV genome with two SeV-Cas9 viruses will prevent HCMV from replicating and forming infectious virions. We measured edited HCMV samples for infectious unit (IU) and genome copy per millilitre. Similar to what has been previously reported, we found a 1 to 1,000 genome copies to IU ratio for TB40/E grown in the absence of a SeV-Cas9 co-infection (Fig. 4d) [46]. The presence of a non-targeting SeV-Cas9 vector did not significantly alter the IU to genome copy ratio of TB40/E, nor did targeting HCMV with a single SeV-Cas9. In fact, the IU to genome copy ratio remained unaltered even when HCMV was targeted with both SeV-Cas9 UL128g1 and UL130g1 viruses (Fig. 4c, d). Deleting this 660+ bp region of the HCMV genome does not inhibit HCMV from replicating and producing virions that can infect fibroblasts. Like the UL128g1 and UL130g1 single-edited viruses, dual-edited HCMV is greatly reduced in its ability to infect epithelial cells (Fig. 4e). Taken together, these data indicate that editing HCMV with SeV-Cas9 does not alter the baseline genome to infectivity ratio for HCMV, even if the genome is edited in multiple places.

### SeV-Cas9 can edit HCMV directly in epithelial and endothelial cells

A major limitation of BAC recombineering is the reliance on fibroblasts. Once the recombinant HCMV genome has been cloned, the BAC is purified and transfected into fibroblasts to amplify infectious virus for characterization [8]. However, it is well known that growing HCMV in fibroblasts causes the virus to acquire mutations, specifically in the PC [12,47]. This can complicate viral tropism and latency studies, which are conducted in epithelial, endothelial and monocytes. An alternative method to produce recombinant HCMV without requiring the passage of the virus in fibroblasts would circumvent this issue.

**Fig. 5.**
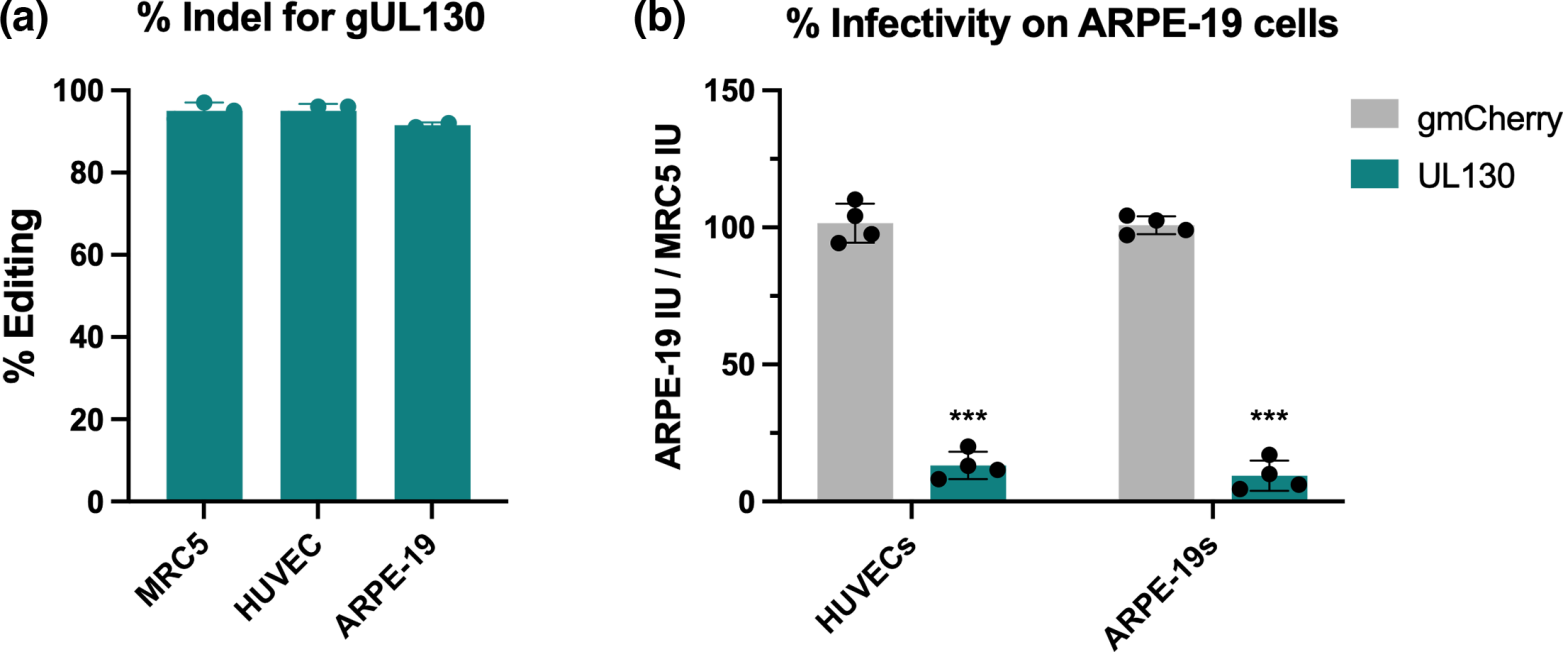
SeV-Cas9 efficiently edits HCMV in epithelial and endothelial cells. (**a**) MRC5 fibroblasts, HUVEC endothelial cells or ARPE-19 epithelial cells were infected with SeV-Cas9 viruses targeting UL130 and then infected with HCMV 2 days later. HCMV viral DNA (vDNA) was extracted 8 dpi and analysed for editing. The percentage of Indels was calculated for three independent experiments. (**b**) Edited HCMV virions from HUVECs and ARPE-19s were titrated on MRC5 fibroblasts and ARPE-19 epithelial cells. The per cent infectivity of each sample was normalized to the non-edited HCMV control. Data are from three independent experiments, and statistical significance was calculated using a two-way ANOVA. T-test with *P*<0.0001.

We sought to determine if our SeV-Cas9 system could edit HCMV directly in relevant cell types like endothelial and epithelial cells. Using the co-infection approach, we evaluated the ability of SeV-Cas9 viruses to edit HCMV in MRC5 fibroblasts, HUVECs and ARPE-19 epithelial cells. In all cell types, SeV-Cas9 editing of UL130 occurred in more than 90% of viral sequences (Fig. 5a). Edited HCMV harvested from both HUVECs and ARPE-19 cells showed a similar cell tropism profile to edited HCMV from MRC5s – virions containing edited UL130 were significantly reduced in their capacity to infect epithelial cells, while virions exposed to the control SeV-Cas9 expressing gmCherry were able to infect epithelial cells at vector control levels (Figs2c  5b). Overall, these experiments demonstrate that HCMV can be successfully edited by SeV-Cas9 directly in epithelial and endothelial cells, cell types of physiological importance.

## Discussion

### Constructing mutant herpesviruses

The ability to manipulate viral genomes and generate recombinant viruses is an essential tool for understanding viral function at the molecular level. HCMV, with the largest genome among human viruses (~236 kb), is estimated to contain anywhere between 200 and 750 ORFs [48,50]. The adoption of BAC recombineering revolutionized herpesvirus genetics, enabling the cloning of large vDNA genomes and facilitating the construction of specific viral mutants [8,48,50]. However, BACs are not without limitations. For example, each strain of HCMV must be engineered independently into a BAC. To date, there are only about ten strains of HCMV that have an available BAC system [9]. Other limitations include the removal of ‘non-essential genes’ to accommodate the BAC cassette, the requirement of passaging the BAC through fibroblasts to grow recombinant virus, the time-consuming nature of these methods and the possibility of off-target modifications [10,13].

The discovery of CRISPR presented new possibilities for creating mutant herpesviruses. Virologists initially targeted essential viral genes, such as IE1/IE2, with CRISPR/Cas9 in attempts to halt viral replication [51,57]. While many groups focused on disrupting ORFs by creating INDELS, several studies have demonstrated the feasibility of adding genetic markers to herpesvirus genomes via homology-directed repair, albeit to lower efficiency [53,58, 59]. For instance, one group targeted the PC of guinea pig cytomegalovirus (GPCMV) in a proof-of-principle experiment akin to ours, showing that knocking out the PC by CRISPR/Cas9 did not limit GPCMV replication in fibroblasts [60]. Furthermore, several groups have engineered cells to express Cas9. This includes a study by Hein and Weissman, where they effectively tiled the entire HCMV genome with gRNAs to disrupt ORFs using Cas9-expressing fibroblasts [61]. In these experiments, Cas9 and gRNAs were delivered by transfection or lentivirus transduction, limiting the types of cells that could be used for Cas9-mediated editing of herpesviruses. Further, Finkel *et al*. engineered HCMV to express single-guide RNA libraries targeting host genes and infect cells expressing Cas9 found in numerous factors that limit virus propagation [62].

### SeV as a vector

SeV serves as a unique vector, offering numerous benefits compared to other viral vectors like lentiviruses or AAVs. First, SeV exhibits broad cell tropism since it uses the ubiquitous sialic acid as its cellular receptor [14]. Second, the pleomorphic virion structure of SeV allows for larger insertions into the viral genome, unlike AAVs [16,17]. This makes it feasible to add the entire enzymes like Cas9 and separate gRNA cassettes. In fact, SeV has been used as a vector to deliver the Yamanaka factors to create induced pluripotent stem cells [63]. Third, because SeV is an RNA virus with no DNA intermediate and replicates entirely in the cytoplasm, there is minimal risk of viral integration into the host genome; this also extends to the HCMV genome. Additionally, mutations that render SeV *ts* allow for restriction and clearance of the virus when non-permissive temperatures are used [20].

A limiting factor for using SeV is the cloning and production of recombinant virus. However, our lab’s optimized reverse genetic system has helped to overcome these barriers [18,20,32]. In the first iteration of our SeV-Cas9 vector, we engineered the virus to target the host gene CCR5 for editing, and we observed editing efficiencies between 75 and 90% [32]. We saw even higher levels of editing of the HCMV genome with SeV-Cas9, with some experiments reaching 95% editing (Figs2b  5a). Notably, the editing efficiency of the SeV-CRISPR/Cas9 system was time-dependent, achieving ~50% editing at 4 dpi, increasing to 75% at 6 dpi and surpassing 90% by 8 dpi (Fig. S1). Importantly, because SeV-CRISPR/Cas9 activity is strictly limited to initial infection stages due to temperature sensitivity, editing occurs exclusively prior to HCMV genome replication. One explanation for why the editing of HCMV is even higher than the host genome is that the viral genome of HCMV is easily accessible to the Cas9/gRNA as soon as the genome is delivered to the nucleus [32]. Also, the slow replicative cycle of HCMV likely makes it easier for the Cas9/gRNA to edit the incoming HCMV genome before viral replication commences, ensuring that all subsequent genomes replicating from that cell would contain the edits. The second iteration of our SeV-Cas9 vector included *ts* mutations that also render the virus relatively IFN-silent [20]. This allows us to deliver the Cas9/gRNA and effectively clear the *ts* SeV-Cas9 by incubating the cells at a non-permissive temperature (37 °C). Additionally, the *ts* SeV-Cas9 vector does not stimulate an IFN response, which made the co-infection with HCMV possible (Fig. 1b, c) and allowed for editing of CCR5 in primary cells like monocytes and CD34+ HSCs. The timeline for designing sgRNAs, generating recombinant SeV vectors and performing HCMV genome editing is outlined in Fig. S2. Compared to lentiviral-based CRISPR systems previously reported, our SeV-based platform achieves more rapid and reproducible editing, with the entire process – from sgRNA design to viral genome editing and analysis – completed within ~21 days. A comparison of the relevant metrics is shown in Table S2. We also systematically assessed potential off-target effects using the CRISPR Design Tool [64]. Computational predictions indicated no significant off-target activities within the human genome for the gRNAs used in this study, even allowing for multiple mismatches. Additionally, sequence analyses via blast against the entire HCMV genome confirmed exclusive specificity for the intended viral targets. Altogether, these details underscore both the precision and practical advantages of our SeV-Cas9 editing platform, enhancing its utility for broader applications.

### Expanding the scope of HCMV recombineering with SeV-Cas9

In this paper, we showed that SeV-Cas9 could edit the HCMV genome and generate recombinant viruses with remarkable efficiency, in a BAC-independent manner. As proof of principle, we targeted the HCMV PC because we could anticipate the altered phenotype of the virus upon disruption of a complete PC. Interestingly, editing HCMV directly in non-fibroblast cells (e.g. endothelial and epithelial cells) may reveal unique functions of both essential and non-essential genes generating unexpected discoveries underscoring the value of the SeV-Cas9 to identify viral factors that contribute to virus replication and spread. Additionally, because editing efficiency progressively increases during infection (50%–90% from 4 to 8 dpi), the presence of WT virus genomes early on may facilitate trans-complementation, allowing isolation of mutants deficient in essential viral genes. This feature notably enhances the utility of our SeV-based system, potentially enabling the recovery of replication-deficient mutants that would otherwise be impossible to propagate from a BAC-derived viral genome. Moreover, the flexibility of our system to edit HCMV in epithelial and endothelial cells also offers an exciting opportunity to investigate cell-type-specific roles of essential genes.

Targeting HCMV in monocytes and CD34+ HSCs with SeV-Cas9 could enhance our understanding of viral latency. Despite recent advancements, the mechanisms underlying HCMV latency establishment, maintenance and reactivation remain elusive, in part because working with HCMV in latency models can be challenging [5,65, 66]. Several HCMV miRNAs, lncRNAs, US28 and the UL135-UL138 gene block of HCMV play pivotal roles in the balance between latency and reaction in monocytes and HSCs [67,72]. These functions were determined by generating mutant viruses by BAC recombineering or lentivirus-delivered CRISPR/Cas9 and initially growing the viruses in fibroblasts. In contrast, the SeV-Cas9 system could enable targeting of the latent HCMV episome for editing in monocytes or HSCs, following the establishment of short-term latency with WT HCMV. This approach could uncover HCMV latency reactivation factors that would be overlooked by other methods.

The potential to engineer HCMV using SeV vectors extends beyond SeV-Cas9. Newer iterations of base editors, such as Prime Editor, can be incorporated into our SeV vector to facilitate directed mutations in the HCMV genome [73,74]. This approach could enable precise insertions of stop codons or other aa changes in HCMV, as opposed to relying on ORF disruption with the traditional Cas9 method. Furthermore, we could use the SeV-Cas9 platform to study differential gene requirements between HCMV strains, a phenomenon which has been noted for UL84 but has been difficult to study using BACs [75]. By using SeV-Cas9 and a conserved gRNA, we could target the same gene in every strain of HCMV, including clinical isolates of HCMV that are not built into a BAC system. Ultimately, we hope to use the SeV-Cas9 system to target additional herpesviruses, providing a new method for mutating diverse herpesviruses beyond the BAC as well as other DNA viruses.

## Supplementary material

10.1099/jgv.0.002126 Uncited Supplementary Material 1.

## Abbreviations

AAV: adeno-associated virus
BAC: bacterial artificial chromosome
CPE: cytopathic effect
DMEM: Dulbecco’s Modified Eagle’s Medium
GPCMV: guinea pig cytomegalovirus
gRNA: guide RNA
HCMV: human cytomegalovirus
HDV: hepatitis D virus
HSC: haematopoietic stem cell
HUVEC: human umbilical vein endothelial cell
IP: immunoprecipitate
IU: infectious unit
IU ml^−1^: infectious unit per millilitre
PBST: phosphate bufferred saline with tween
PC: pentamer complex
qPCR: quantitative polymerase chain reaction
SeV: Sendai virus
*ts*: temperature-sensitive
vDNA: viral DNA
