# Supplementary material for: Precision engineering of human cytomegalovirus without BAC constraints: a Sendai virus-delivered CRISPR/Cas9 approach
Source: J Gen Virol. 2025 Jul 15;106(7):002126. doi: 10.1099/jgv.0.002126 (PMC12311846; doi:10.1099/jgv.0.002126)

Table S1 (Part 1). List of primer and gRNA sequences used for cloning in this study. Names, purpose and primer sequence are indicated.

**Cloning UL128 g1 SeV-Cas9**	**UL128 g1 (exon1):**	**CTGCGCGTACCCGCGGCACG**
**Name of Primer**	**Purpose of primer**	**Primer sequence**
JC007	Forward primer to amplify the gRNA to UL128 guide 1	TAACACGGCGCAGCGATCGCGCGCAGCTGATGAGTCCGTGAGGA
JC008	Reverse primer to amplify the gRNA to UL128 guide 1	CGTGCCGCGGGTACGCGCAGGACGGTAGGAATTCCTACC
JC009	Forward primer to amplify the gRNA and tracrRNA against UL128 guide 1	GCACGGTTTTAGAGCTAGAAATAGCAAGTTAAAATAAG
HDV-SnaBI-R	Universal reverse primer for two step PCR for SeV-Cas9 gRNA cloning	CCTCACCCGGGATACGTAG

**Cloning UL128 g2 SeV-Cas9**	**UL128 g2 (exon2):**	**CTGTAGCAGACTTCGCCGTC**
**Name of Primer**	**Purpose of primer**	**Primer sequence**
JC010	Forward primer to amplify the gRNA to UL128 guide 2	TAACACGGCGCAGCGATCGCCTACAGCTGATGAGTCCGTGAGGA
JC011	Reverse primer to amplify the gRNA to UL128 guide 2	GACGGCGAAGTCTGCTACAGGACGGTAGGAATTCCTACC
JC012	Forward primer to amplify the gRNA and tracrRNA against UL128 guide 2	CCGTCGTTTTAGAGCTAGAAATAGCAAGTTAAAATAAG
HDV-SnaBI-R	Universal reverse primer for two step PCR for SeV-Cas9 gRNA cloning	CCTCACCCGGGATACGTAG

**Cloning UL130 g1 SeV-Cas9**	**UL130 g1:**	TTCGCCGTTAGCGTTGACCA
**Name of Primer**	**Purpose of primer**	**Primer sequence**
JC013	Forward primer to amplify the gRNA to UL130 guide 1	TAACACGGCGCAGCGATCGCGGGTTCCTGATGAGTCCGTGAGGA
JC014	Reverse primer to amplify the gRNA to UL130 guide 1	CCTTGCAATTCTCGGGGTTCGACGGTAGGAATTCCTACC
JC015	Forward primer to amplify the gRNA and tracrRNA against UL130 guide 1	GCAAGGGTTTTAGAGCTAGAAATAGCAAGTTAAAATAAG
HDV-SnaBI-R	Universal reverse primer for two step PCR for SeV-Cas9 gRNA cloning	CCTCACCCGGGATACGTAG

**Cloning UL130 g2 SeV-Cas9**	**UL130 g2:**	GAACCCCGAGAATTGCAAGG
**Name of Primer**	**Purpose of primer**	**Primer sequence**
JC016	Forward primer to amplify the gRNA to UL130 guide 2	TAACACGGCGCAGCGATCGCGGCGAACTGATGAGTCCGTGAGGA
JC017	Reverse primer to amplify the gRNA to UL130 guide 2	TGGTCAACGCTAACGGCGAAGACGGTAGGAATTCCTACC
JC018	Forward primer to amplify the gRNA and tracrRNA against UL130 guide 2	GTTGACCAGTTTTAGAGCTAGAAATAGCAAGTTAAAATAAG
HDV-SnaBI-R	Universal reverse primer for two step PCR for SeV-Cas9 gRNA cloning	CCTCACCCGGGATACGTAG

**Cloning US2 g1 SeV-Cas9**	**US2 g1:**	AACCGGTGCTACATTGACAA
**Name of Primer**	**Purpose of primer**	**Primer sequence**
JC050	Forward primer to amplify the gRNA to US2 guide 1	TAACACGGCGCAGCGATCGCCCGGTTCTGATGAGTCCGTGAGGA
JC051	Reverse primer to amplify the gRNA to US2 guide 1	TTGTCAATGTAGCACCGGTTGACGGTAGGAATTCCTACC
JC052	Forward primer to amplify the gRNA and tracrRNA against US2 guide 2	ACATTGACAAGTTTTAGAGCTAGAAATAGCAAGTTAAAATAAG
HDV-SnaBI-R	Universal reverse primer for two step PCR for SeV-Cas9 gRNA cloning	CCTCACCCGGGATACGTAG

**Cloning US2 g2 SeV-Cas9**	**US2 g2:**	AAGGCCGATTATGGCGGCGT
**Name of Primer**	**Purpose of primer**	**Primer sequence**
JC053	Forward primer to amplify the gRNA to US2 guide 2	TAACACGGCGCAGCGATCGCGGCCTTCTGATGAGTCCGTGAGGA
JC054	Reverse primer to amplify the gRNA to US2 guide 2	ACGCCGCCATAATCGGCCTTGACGGTAGGAATTCCTACC
JC088	Forward primer to amplify the gRNA and tracrRNA against US2 guide 2	GCCGATTATGGCGGCGTGTTTTAGAGCTAGAAATAGCAAGTTAAAATAAG
HDV-SnaBI-R	Universal reverse primer for two step PCR for SeV-Cas9 gRNA cloning	CCTCACCCGGGATACGTAG

**Cloning US11 g3 SeV-Cas9**		
**Name of Primer**	**Purpose of primer**	**Primer sequence**
JC062	Forward primer to amplify the gRNA to US11 guide 3	TAACACGGCGCAGCGATCGCCCCAACCTGATGAGTCCGTGAGGA
JC063	Reverse primer to amplify the gRNA to US11 guide 3	GGACTATCGTGTTCCCCAACGACGGTAGGAATTCCTACC
JC090	Forward primer to amplify the gRNA and tracrRNA against US11 guide 3	CGTTGGGGAACACGATAGTCCGTTTTAGAGCTAGAAATAGCAAGTTAAAATAAG
HDV-SnaBI-R	Universal reverse primer for two step PCR for SeV-Cas9 gRNA cloning	CCTCACCCGGGATACGTAG

Table S1 (Part 2). List of primer and gRNA sequences used for cloning in this study. Names, purpose and primer sequence are indicated.

**UL128 sequencing primers**		
**Name of Primer**	**Purpose of primer**	**Primer sequence**
JC029	Forward UL128 amplifying primer	GATTTTTCCAATATCGCCATCTCTATCGG
JC030	Reverse UL128 amplifying primer	TTCAGGTGCGATTGACGTTCAC
JC031	Forward UL128 sequencing primer	CCAGACTTACACCTTCTGCAC

**UL130 sequencing primers**		
**Name of Primer**	**Purpose of primer**	**Primer sequence**
JC032	Forward UL130 amplifying primer	TAACAGCCACAACGCCGTCAA
JC033	Reverse UL130 amplifying primer	CGACTTTAGACGTCAGAACCGT
JC034	Forward UL130 sequencing primer	ACCAACAAAAGGACCACG

**US2 sequencing primers**		
**Name of Primer**	**Purpose of primer**	**Primer sequence**
JC095	Forward US2 primer	CACTAAAGCCGGGGAAGACG
JC096	Reverse US2 primer	GCCAGATCACATCCCCTTGCAG

**US11 sequencing primers**		
**Name of Primer**	**Purpose of primer**	**Primer sequence**
JC097	Forward US11 primer	AGCCTTACAGCTTTTGAGTCTAGACAG
JC098	Reverse US11 primer	ACGAGCTCCACAGGGGAAC

**Primers to TA clone HCMV UL83 into pGEM-T**		
**Name of Primer**	**Purpose of primer**	**Primer sequence**
JC105	Forward amplifying primer for UL83	ACACAACACCGTAAAGCCG
JC106	Reverse amplifying primer for UL83	CCCATGTCGATCTATGTGTACG

**qPCR primers for UL83**		
**Name of Primer**	**Purpose of primer**	**Primer sequence**
JC107	Forward qPCR primer for UL83	TGGTCACCTATCACCTGCAT
JC108	Reverse qPCR primer for UL83	GAAAGAGCCCGACGTCTACT

Table S2. Kinetics and editing efficiencies of SeV-CRISPR/Cas versus lentiviral-mediated CRISPR/Cas editing of HCMV genomes.

Reference	HCMV target	CRISPR/Cas Vector used for HCMV genome editing	Cell line	HCMV MOI	Time point analyzed	Substrate analyzed	Assay used	Editing Efficiency
Carmichael et al, 2025 (This study)	UL128, UL130, US2	SeV-CRISPR/Cas	MRC5	0.1	8 dpi	Virus	Amplicon sequencing, ICE anlaysis	>90%
			APRE19	0.1	8 dpi			>90%
			HUVEC	0.1	8 dpi			>90%
van Diemen et al, 2016	US6, US7	pSicoR-CRISPR-PuroR and/or pSICOR-CRISPR-BlastR	MRC5	0.05	2 dpi	Virus	454 sequencing	~80%
	UL54, UL70		MRC5	0.5	21 dpi			1.3 - 4.6%[a]
Fu et al, 2017	UL82	lentiCRISPRv1	HFF	N.A.	20 dpi	N.A.	PCR screening	N.D.[b]
Gergen et al, 2018	U122/123	LV (type 1) Cas9-T2A-PuroR	MRC5	0.1	2 dpi	infected cell lysate	T7E1	29%
			MRC5	0.1	2 dpi		T7E1	5.4%[c]
		LV (type 2) Cas9-HAtag-GFP	U-251 MG[d]	0.1	8 dpi		T7E1	30-50%
King and Munger, 2019	GFP KO in HCMV-EGFP	pLentiCRISPRv2	MRC5	0.25	10 dpi	Virus	GFP(-) plaques	80%
Xiao et al, 2020	IE	pLentiCRISPRv2	HFF	1	3 dpi (72 hpi)	infected cell lysate	T7E1	50%
	IE	pLentiCRISPRv3	THP1	10	3, 5, 7 days pre- and post-TPA differentiation	N.D.	N.D.	N.D.[e]

[a] Due to outgrowth and selection of functional in-frame deletions.

[b] UL82-deficient virus was made successfully but no data provided on editing efficiency.

[c] Multiplex (2 gRNAs) editing efficiency versus 29% for singleplex.

[d] Formerly known as U-373.

[e] Editing efficiency was not monitored; only decreases in UL 122, UL54 and UL83 transcripts were monitored pre- and post- TPA differentiation.

Figure S1. SeV-Cas9 mediated editing of UL128. Samples from SeV-Cas9 UL128 g1 and HCMV co-infected cells were harvested at 4, 6, and 8 dpi with HCMV. Viral DNA was extracted, sequenced, and analyzed with ICE for the indicated time points (A) and samples were titrated on MRC5 fibroblasts and ARPE-19 epithelial cells (B). The percent infectivity of each sample was normalized to the non-edited (No SeV) HCMV control (C). Data are from 3 independent experiments.

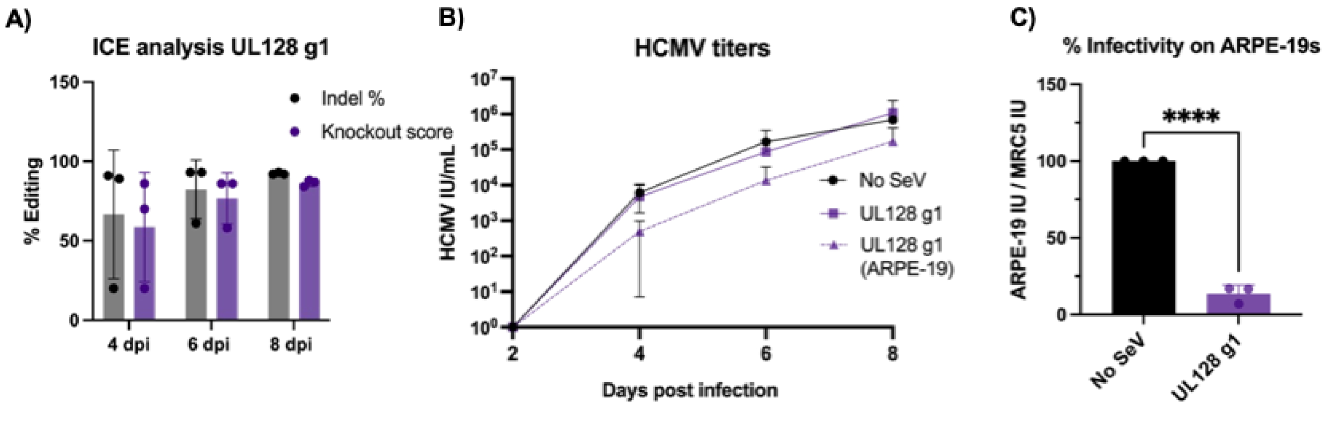

Figure S2. Timelines for SeV-CRISPR/Cas versus lentiviral-mediated CRISPR-Cas editing of HCMV. Timelines were extracted from the methods section of the indicated references when specifically stated. Asterisks (*) indicate best estimates if not specifically stated (based on typical times from other studies). Green and red arrowheads represent initial day of HCMV infection and day of analysis for HCMV genome editing, respectively. PCR & cloning were all standardized to 2 days. Editing efficiencies are shown in Table S2.

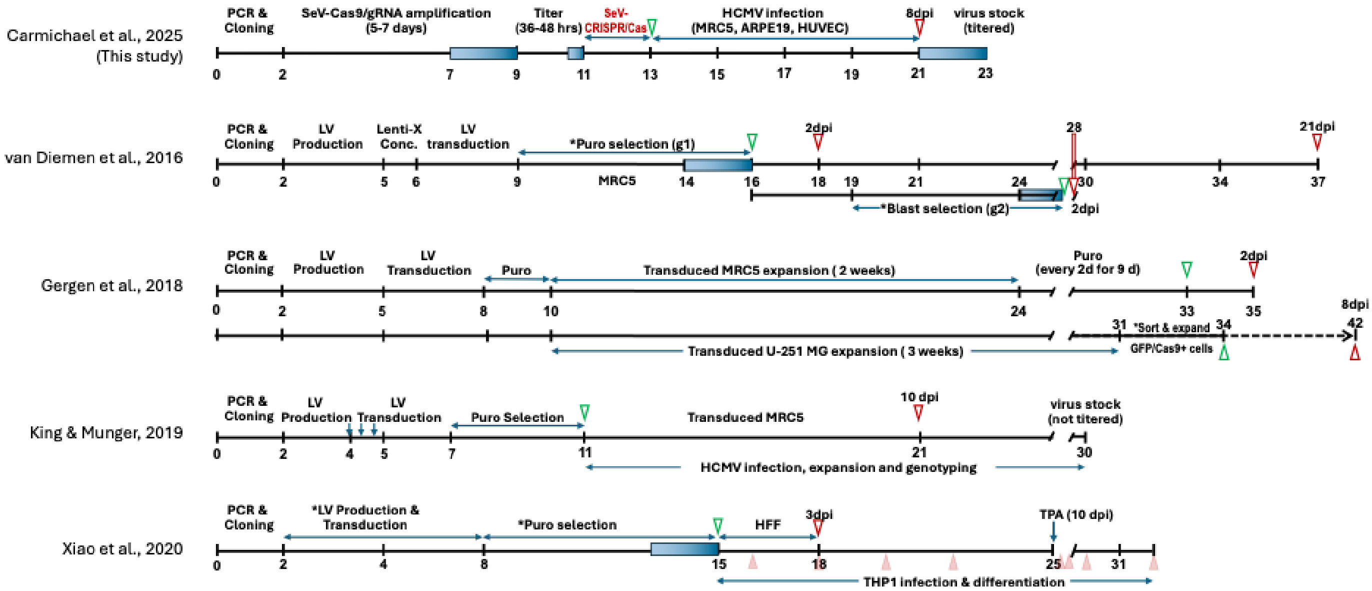

Supplement: Uncited Supplementary Material 1. [file jgv-106-02126-s001.pdf]
